# Quadrupling the capacity of post aerobic digestion treating anaerobically digested sludge using a moving-bed biofilm (MBBR) configuration

**DOI:** 10.1016/j.wroa.2024.100240

**Published:** 2024-07-23

**Authors:** Zhiyao Wang, Xi Lu, Min Zheng, Zhetai Hu, Damien Batstone, Zhiguo Yuan, Shihu Hu

**Affiliations:** a Australian Centre for Water and Environmental Biotechnology (ACWEB, formerly AWMC)^,^ The University of Queensland^,^ St. Lucia^,^ Queensland 4072^,^ Australia; b Water Research Centre, School of Civil and Environmental Engineering, University of New South Wales, Sydney, New South Wales 2052, Australia; c School of Energy and Environment^,^ City University of Hong Kong^,^ Hong Kong SAR^,^ China

**Keywords:** Acidic aerobic sludge digestion, *Candidatus* Nitrosoglobus, Acid-tolerant ammonia oxidizers, Free nitrous acid

## Abstract

Wastewater treatment plants produce large amounts of sludge requiring stabilization before safe disposal. Traditional biological stabilization approaches are cost-effective but generally require either an extended retention time (10–40 days), or elevated temperatures (40–80 °C) for effective pathogens inactivation. This study overcomes these limitations via a novel acidic aerobic digestion process, leveraging an acid-tolerant ammonia-oxidizing bacterium (AOB) *Candidatus* Nitrosoglobus. To retain this novel but slowly growing AOB, we proposed the first-ever application of a classical wastewater configuration—moving bed biofilm reactor (MBBR)—for sludge treatment. The AOB in biofilm maintains acidic pH and high nitrite levels in sludge, generating free nitrous acid in situ to expedite sludge stabilization. This process was tested in two laboratory-scale aerobic digesters processing full-scale anaerobically digested sludge. At an ambient temperature of 20 °C, pathogens were reduced to levels well below the threshold specified for the highest stabilization level (Class A), within a retention time of 3.5 days. A high volatile solids reduction of 27.4 ± 5.2% was achieved. Through drastically accelerating stabilization and enhancing reduction, this process substantially saves capital and operational costs for sludge disposal.

## Highlights

• MBBR was used to treat anaerobically digested sludge at an HRT of 3.5 days.
• Acid-tolerant AOB immobilized on carriers generate acidic pH and free nitrous acid.
• The MBBR-based system improved the AD sludge stabilization level to Class A.
• The system effectively reduced solids contents by ∼30% and slightly improved dewaterability.

## Graphical abstract

**Figure:**
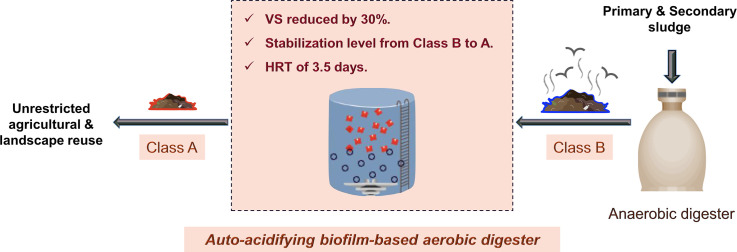

## Introduction

Wastewater treatment plants (WWTPs) generate a massive amount of excess sludge. For safe disposal, sludge needs to be stabilized, which involves reducing the putrescible organic content and pathogens (U.S. Environmental Protection Agency, 1994). Anaerobic digestion is the most extensively used method for sludge stabilization in medium to large-sized WWTPs, as it allows for simultaneous sludge stabilization and energy recovery (Tchobanoglus et al., 2003). However, anaerobically digested (AD) sludge typically only meets the requirements of Class B stabilization level, not Class A (U.S. EPA Part 503 Biosolids Rule) (Boczek et al., 2023). Class B biosolids, due to their higher pathogen contents, are restricted from public access areas, thus requiring longer transport distances and higher costs for disposal (Foess and Fredericks, 1995). To reduce sludge treatment costs and enhance beneficial reuse opportunities, many communities are working towards producing Class A biosolids.

AD sludge can be upgraded to Class A biosolids via various post-treatment options, including physicochemical and biological approaches, or their combinations (Wang et al., 2021a). Physicochemical methods are effective but require substantial energy inputs, such as heat (Svennevik et al., 2019), ultrasound (Song et al., 2017), high pressure treatments (Friedman et al., 1988), and chemicals like peroxide (Abelleira et al., 2012), lime (Brewster et al., 2002), nitrite (Wang et al., 2016), acids (Yu et al., 2022). Biological post-treatment is more cost-effective but less efficient in solids reduction and stabilization, as the residual organics in AD sludge are recalcitrant to biodegradation (Wang et al., 2021a). For instance, post-treatment of AD sludge under aerobic/anaerobic conditions still rarely meets Class A biosolids standards unless combined with thermal treatment (Novak et al., 2011; Parravicini et al., 2008; Tomei et al., 2011; Zhang et al., 2016).

Recently, acidic aerobic post-treatment has been demonstrated as a promising alternative to further reduce and stabilize AD sludge at ambient temperatures (Wang et al., 2021d). The acidic conditions are achieved through the metabolism of an acid-tolerant ammonia oxidizing bacteria (AOB) Candidatus (*Ca.)* Nitrosoglobus (NH_4_^+^ + 1.5O_2_ → NO_2_^-^ + 2H^+^+ 2H_2_O), instead via chemical acid addition. At acidic pH, nitrite (NO_2_^-^) easily accumulates and protonates into free nitrous acid (FNA, HNO_2_) (Meng et al., 2022; Wang et al., 2021e), which significantly accelerates hydrolysis of complex organics in sludge and pathogen inactivation (Jiang et al., 2011). This acidic aerobic post-treatment effectively upgraded AD sludge to Class A stabilization level (Wang et al., 2021d). However, it required a relatively long hydraulic retention time (HRT) of ∼ 15 days to retain the key microorganism *Ca.* Nitrosoglobus (Wang et al., 2021d). This lengthy retention time is prohibitive for the adoption of this technology in WWTPs. The advantages of acidic sludge digestion process, i.e., enabling a higher sludge reduction and stabilization rate facilitated by acidic pH/FNA, have not been translated into a larger capacity.

To overcome this limitation, here we propose a novel process design for sludge treatment. Drawing inspiration from wastewater treatment, where the biomass can be effectively retained using the moving-bed biofilm reactor (MBBR) configuration (Hem et al., 1994), we propose to immobilize the functional microbe *Ca.* Nitrosoglobus in biofilm in the sludge digester, constituting an MBBR acidic aerobic digestion process.

## Results

This study demonstrates the feasibility of rapid sludge stabilization via a novel biofilm-based acidic aerobic digestion process. To this end, we set up and operated two lab-scale acidic aerobic sludge digesters for 14 months to stabilize AD sludge. One digester was inoculated with *Ca.* Nitrosoglobus colonizing in biofilm on plastic carriers, named MBBR digester, while the other was seeded with *Ca.* Nitrosoglobus in suspended sludge (SS), referred to as SS digester (see details of the inocula in Material and Methods). Both digesters were operated under the same conditions, subject to a stepwise decrease of HRT from 15 to 3 days. Table 1 presents the HRTs, VS loading/removal rates, and dissolved oxygen (DO) levels across different phases. We evaluated the sludge stabilization performance by monitoring the levels of total solids (TS), VS, and indicators of pathogenic microorganisms, and the specific oxygen uptake rate (SOUR).

**Table 1:** The operational conditions of the MBBR digesters in six phases.

	Phase Ⅰ	Phase Ⅱ	Phase Ⅲ	Phase Ⅳ	Phase Ⅴ	Phase Ⅵ
Period	0–30	31–118	119–210	211–316	317–380	381–450
Hydraulic retention time (HRT) (days)	15	10	7.5	5	3.5	3
Volume of sludge exchange each day (mL)	100	150	200	300	429	500
Volatile solids (VS) loading rates (kg/m^3^/d)	1.3	2.0	2.7	4.0	5.7	6.7
VS reduction rates (kg/m^3^/d)	0.4	0.6	0.7	0.8	1.2	0.4
Dissolved oxygen (mg/L)	7.2 ± 0.5	6.5 ± 0.3	5.8 ± 0.4	5.0 ± 0.4	3.5 ± 0.3	0.5 ± 0.3

### Establishment and maintenance of auto-acidifying conditions

**Fig. 1:**
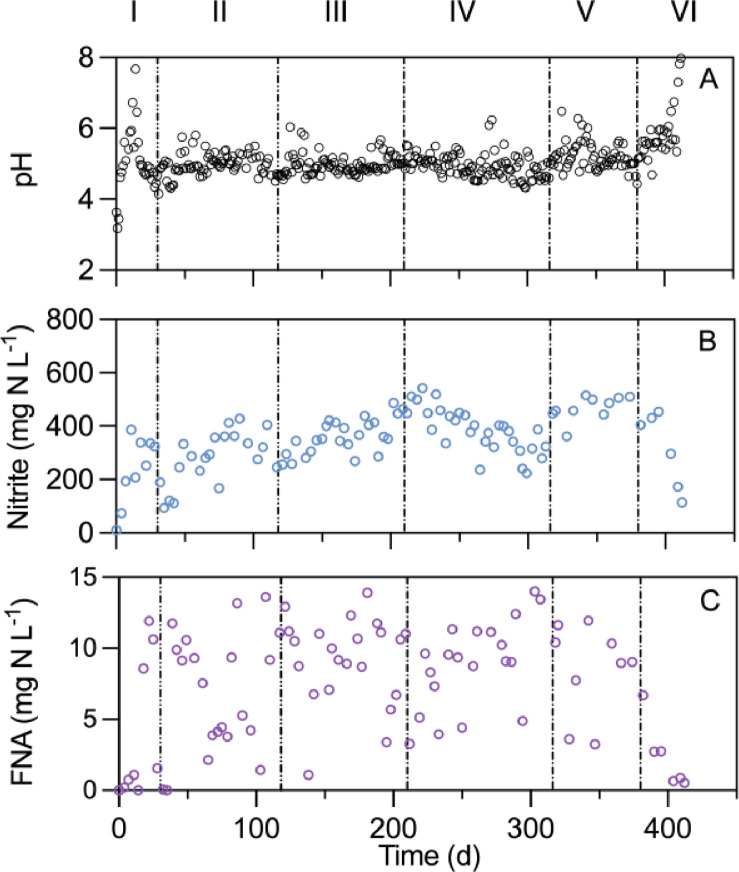
The temporal profiles of (A) pH; (B) nitrite; and (C) FNA levels in the aerobic MBBR sludge digester receiving anaerobically digested sludge. The hydraulic retention time (HRT) was progressively decreased from 15 (I), 10 (II), 7.5 (III), 5 (IV), 3.5 (V), to 3 days (VI).

Both the MBBR and the SS digesters started with an HRT of 15 days in Phase Ⅰ (0–30d). From Day 0 to 15, the pH in the MBBR digesters gradually increased from ∼ 4 to ∼ 7.8 (Fig. 1A) due to the daily feed of AD sludge. Subsequently, the pH rapidly decreased to 4–5, possibly driven by the inoculated acid-tolerant AOB which generates H^+^ via oxidizing NH_4_^+^ (Wang et al., 2021e). Similarly, the pH in the SS digester also rose first and then declined to the slightly acidic range (4–5) within the first 30 days (Fig. 1B). Concomitant with the pH decrease, nitrite gradually accumulated to ∼ 350 mg N L^-1^ in both the MBBR (Fig. 1B) and the SS digesters (Fig. S1B). Such a nitrite concentration at pH 4–5 is estimated to form FNA, in situ, around 10 mg HNO_2_-N L^-1^ (Fig. 1C and Fig. S1C). The in situ acidic pH and high FNA indicated successful establishment of auto-acidifying conditions in both digesters.

From Day 31, the HRT was shortened to 10 days for both digesters. The pH in the SS digester immediately increased to 6 and further to 8 within 20 days (Fig. S1A). As a result of the rising pH, the FNA decreased to < 0.5 mg HNO_2_-N L^-1^ (Fig. S1C), indicating the collapse of auto-acidifying conditions. The rising pH is likely induced by the washout of acid tolerant AOB under a short HRT of 10 days (as confirmed with microbial data, not shown here). Therefore, the SS digester was terminated thereafter. In contrast, the MBBR digester successfully maintained an acidic pH and a stable nitrite concentration (Fig. 1) in Phase Ⅱ, likely owing to the immobilization of *Ca.* Nitrosoglobus on carriers, which will be further discussed in microbial community Section. These results illustrate that the MBBR configuration indeed enables a higher capacity compared to the SS configuration.

To gauge the maximal capacity attainable in the MBBR digester, the HRT was further decreased from 10 days to 7.5 (Phase III), 5 (Phase IV), and 3.5 days (Phase V), corresponding to an increase of VS loading rates from 2.7 in Phase III to 5.7 kg/m^3^/d in Phase V (Table 1). Except for a few occasional pH peaks due to operational issues (e.g., failure of aeration pumps), the pH stabilized around 5 throughout Phase III–V (Fig. 1A). During this period, the nitrite and FNA concentrations were maintained at high levels of ∼ 400 and ∼ 10 mg N L^-1^, respectively (Fig. 1B and C). The MBBR digester collapsed when the HRT was further decreased to 3 days (Phase VI), as indicated by the increasing pH and plummeting nitrite/FNA (Fig. 1).

The final collapse was likely due to the decreasing DO concentration (Table 1). As the VS loading increased from Phase Ⅰ to VI, the oxygen consumption rates rose. However, the oxygen supply rate remained constant, as all phases adopted the same air flowrate (3.5 L/min), the maximum achievable with the laboratory air pump without causing uncontrollable foaming issue. Consequently, the DO decreased from Phase Ⅰ (7.2 ± 0.5 mg/L) to VI (0.5 ± 0.3 mg/L) (Table 1). The low DO concentration in Phase VI restricted the oxygen available to AOB in the biofilm, leading to the observed rising pH and the sharp decrease in nitrite and FNA (Fig. 1). Collectively, the capacity of the MBBR digester quadrupled compared to the SS digester.

**Fig. 2:**
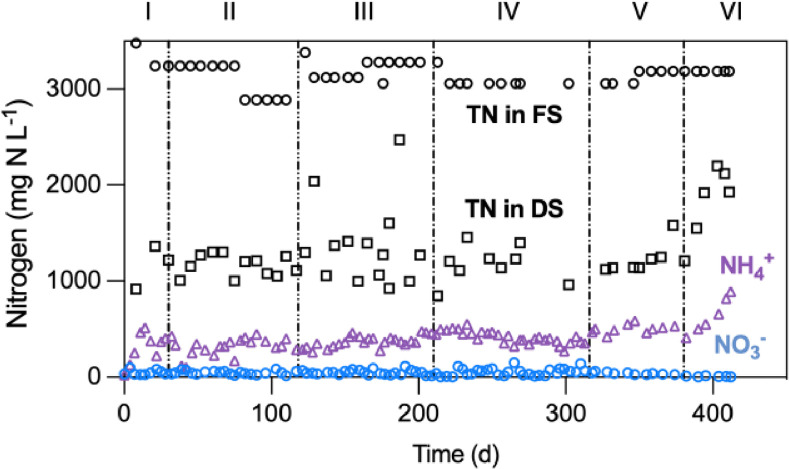
Temporal profiles of nitrate (NO_3_^-^, blue solid circles), ammonium (NH_4_^+^), purple triangles), and the total nitrogen (TN = Kjeldahl + nitrite + nitrate nitrogen) concentrations in the discharged sludge (DS, black squares) from the MBBR digester. TN in the feed sludge (FS, black circles) is also shown.

The NH_4_^+^ concentration in the MBBR digester decreased from about 1200 mg N L^-1^ (feed sludge, Table S1) to 352 ± 34 mg N L^-1^ (digester, Fig. 2) throughout Phase I–V, indicating the occurrence of nitrification. The product of nitrification was dominated by NO_2_^-^ instead of NO_3_^-^ (below 50 mg N L^-1^, Fig. 2) over the whole study. The marginal NO_3_^-^ concentration indicated that nitrite-oxidizing bacteria (NOB) were suppressed in the MBBR digester, which was likely due to the high in situ FNA concentration of ∼ 10 mg N L^-1^ (Fig. 1C) (Wang et al., 2021e). In Phase VI, the NH_4_^+^ concentration significantly increased to > 700 mg N L^-1^, concurring with the pH rise in Phase VI (Fig. 1A). The increase of NH_4_^+^provided direct evidence that the AOB activity severely declined in Phase VI which was likely induced by the low DO concentrations (Table 1).

Notably, the TN concentration decreased from 3400 ± 130 mg N L^-1^ (feed sludge) to 1125 ± 86 mg N L^-1^ (discharged sludge) (Fig. 2) during Phase I–V, signifying significant TN loss. The off gases of the MBBR digester were monitored for the quantification of nitric oxide (NO) and nitrous oxide (N_2_O) emissions (Fig. S3). These emissions accounted for 3.8 ± 0.5% and 5.2 ± 0.7% of the TN loss, or 1.3% and 2.7% of the influent N load, respectively, with the remaining loss (∼90%) presumably being N_2_. This significant N loss was most likely induced by denitrification, occurring in the inner zones of biofilm or thick flocs where oxygen was deficient due to oxygen transfer limitations (Li and Bishop, 2004).

### Sludge reduction and stabilization

**Fig. 3:**
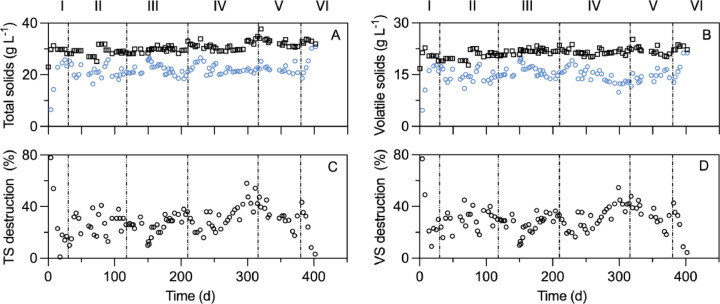
The TS (A) and VS (B) concentrations in the feed sludge (FS, black squares) and discharged sludge (DS, blue circles) from the MBBR digester; the TS (C) and VS (D) destruction efficiencies of the MBBR digester.

The MBBR digestor reduced the TS and VS concentrations from 28.9 ± 0.9 and 20.1 ± 3.7 g l^-1^ in the feed AD sludge to 21.0 ± 3.2 and 14.5 ± 1.7 g l^-1^, respectively (Fig. 3A and B), giving rise to a TS and VS destruction efficiency of 27.9 ± 4.3% and 27.4 ± 5.2%. The TS and VS destruction efficiencies did not decrease in Phase Ⅱ when HRT was shortened to 10 days. In contrast, the TS and VS reduction efficiency of the SS digester, despite being comparable to the MBBR digester in Phase I, significantly (*p* < 0.05) decreased to 17.1 ± 2.1% and 15.8 ± 3.2%, respectively, in Phase Ⅱ (Fig. S2). The distinct performance between the two digesters in Phase Ⅱ is likely due to sharply different in situ pH and FNA conditions (Fig. 1 and Fig. S1). In the MBBR digester, pH and FNA maintained at the same levels as in Phase I (Fig. 1), which was not the case in the SS digester (Fig. S1).

**Fig. 4:**
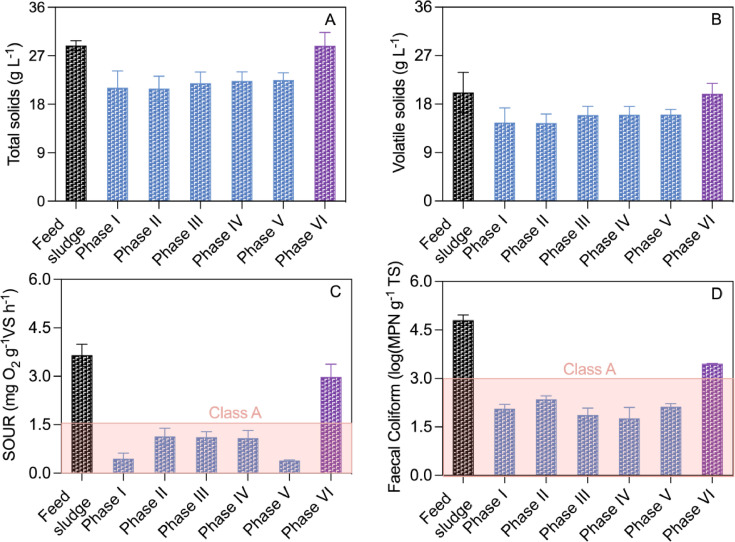
The average TS (A), VS (B), SOUR (C), and *Faecal Coliforms* (D) levels in the feed sludge and the MBBR sludge across Phase I–VI. SOUR and pathogens were compared against Class A biosolids threshold specified by U.S. EPA. Error bars represent standard deviations.

The TS and VS concentrations in the MBBR digester slightly increased at the beginning of each of Phases II-IV, but quickly dropped to a level comparable to that in Phase I (Fig. 4A and B). The transient rises were due to dynamics caused by the sharp rises in the organic loading rate at the beginning of each phase (Table 1). Notably, in Phase V, the MBBR digester was receiving sludge at a rate 4.3 times that in Phase I, yet a commensurate solids reduction efficiency was maintained. In Phase VI, the TS and VS destruction dropped to negligible levels, concomitant to a sharp pH rise to 8.0 and a sharp FNA drop to nearly 0 (Fig. 1).

Akin to the solids destruction efficiency, the sludge stabilization performance of the MBBR digester remained consistent in Phases I-V (Fig. 4C and D). *Faecal Coliforms* abundance, an indicator of pathogens) were reduced by over 2 logs from 4.8 ± 0.2 log (MPN g^-1^TS) in the feed sludge to 2.1 ± 0.3 log (MPN g^-1^TS). Similarly, the SOUR decreased from 3.7 ± 0.7 to 0.3–1.1 mg O_2_ g^-1^VS h^-1^. Both *Faecal Coliforms* and SOUR levels met Class A biosolids criteria (U.S. Environmental Protection Agency, 1994). The stabilization performance declined in Phase VI due to a sharp increase in pH and a drop in FNA (Fig. 1). The stabilization level of sludge from the SS digester also met Class A level at HRT of 15 days but fell to Class B when HRT decreased to 10 days (Fig. S2).

### Sludge dewaterability

**Fig. 5:**
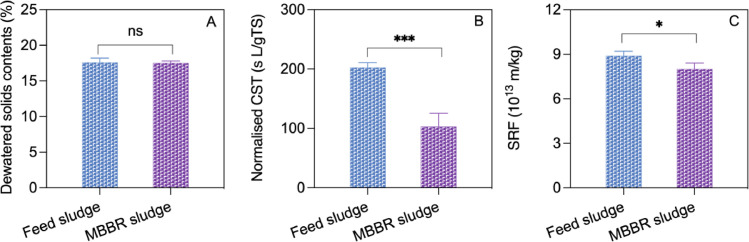
The solids contents of the dewatered sludge cake (A), normalised capillary suction time (CST) (B), and specific resistance to filtration (SRF) (C) in the feed sludge and the MBBR sludge in Phase V. Error bars represent standard deviations (*n* = 6). ns, *, *** indicate *p* > 0.05, *p* < 0.05 and *p* < 0.001, respectively.

In Phase V, the dewaterability of the feed and the MBBR sludges were assessed and compared using three different methods. The solids content of the dewatered cake obtained using centrifugation did not change significantly (*p* > 0.05) after acidic aerobic digestion (Fig. 5A). However, the normalised capillary suction time (CST) (Fig. 5B) and specific resistance to filtration (SRF) (Fig. 5C) were both significantly lower for the MBBR sludge than for the feed sludge, with the *p* value smaller than 0.001 and 0.05, respectively, indicating improved dewaterability after acidic aerobic sludge digestion. The insignificant changes of dewatered solids content could be attributed to the low sensitivity of the method or measurement errors, which might have masked any changes. Additionally, the scale of our laboratory-scale centrifugation might not be sufficient to detect variations in dewaterability.

### Microbial community in the suspended sludge and the biofilm

The microbial community compositions in the MBBR digester were investigated using 16S rRNA gene amplicon sequencing. Both biofilm on the carriers and suspended sludge samples were taken in Phase V. The suspended sludge and the biofilm show a similar community structure at both the Phylum and the Genus levels (Fig. S4 and S5). *Bateroidota, Proteobacteria, Halobacterota, Chloroflexi, Firmicutes*, and *Actinobacteriota* were the most abundant phyla in both the suspended sludge and the biofilm, with the relative abundances above 5%. At the genus level, *Fluviicola* and *Chujaibacter* were the two most dominant genera, concurring with the results in previous acidic systems (Meng et al., 2022; Wang et al., 2021d, 2021e). Only one nitrifying genus, *Ca.* Nitrosoglobus, was identified in the MBBB digester. The relative abundance of *Ca.* Nitrosoglobus was 0.1 ± 0.0% in the suspended sludge and 2.6 ± 0.2% in the biofilm. The presence of *Ca.* Nitrosoglobus in suspended sludge was likely due to the detachment of *Ca.* Nitrosoglobus from biofilms, as the low SRT (3.5 days) of the suspended sludge was too short to retain this microorganism (Wang et al., 2021b).

## Discussion

This study demonstrates that an auto-acidifying biofilm-based aerobic digestion (ABAD) process effectively upgraded AD sludge from Class B to Class A stabilization level and reduced its VS contents by 27.4 ± 5.2%. The sludge reduction and stabilization performance is comparable to previous acidic aerobic post-treatment process. However, this was achieved with an HRT of only 3.5 days compared to 15 days in the previous study (Wang et al., 2021d), quadrupling the sludge treatment capacity. This study, for the first time, overcomes the long retention time limitation of acidic aerobic digestion processes, unleashing its full potential of intensifying sludge treatment system.

Over the past decades, the principal focus in wastewater engineering has been on improving effluent quality. A byproduct of these efforts is the increasing production of sludge, which has been growing at an annual rate of 1% (UNESCO World Water Assessment Programme, 2018). Therefore, it is imperative for the water industry to expand the capacity of sludge treatment facilities. However, biological sludge stabilization, relying on enzyme-catalysed reactions, is inherently slower compared to physio-chemical methods. Many research efforts tried to accelerate biological stabilization through elevating temperatures (a 10 °C rise will increase the activity of most enzymes by 50–100%) (Cao and Wang, 2016), engendering the thermophilic digestion technologies, like thermophilic anaerobic digestion, thermophilic composting, and autothermal aerobic digestion (ATAD) (Zhang et al., 2022). In contrast, this study innovatively sought to expedite biological stabilization via pH alteration, which offers a different perspective of intensifying sludge treatment systems.

The superior performance can be attributed to the cascade effects of MBBR configuration and acidic AOB. MBBR was initially developed for intensifying wastewater treatment and has been widely implemented since the 1990s (Ødegaard, 2006; Ødegaard et al., 1994). By allowing immobilization of biomass on the surface of carriers, MBBR enriches slowly-growing microorganisms, such as nitrifiers, allowing plants to handle increased loadings rates without expansion (Ødegaard, 2006). This study is the first time, to the best of our knowledge, that the MBBR configuration is employed to intensify sludge treatment.

In sludge treatment systems, unlike wastewater systems, retaining biomass not necessarily allows for process intensification, as biomass retention is not the rate-limiting step. Instead, the bottleneck is sludge hydrolysis and pathogen inactivation (Gonzalez et al., 2018). Here, MBBR carriers enables effective retention of acid tolerant AOB, which generates acidic pH and FNA; while acidity/FNA directly promotes capacity elevation by accelerating sludge hydrolysis and pathogen inactivation (Wang et al., 2021d). In future, it is worthwhile to further investigate the potential of MBBR to intensifying other biological sludge treatment systems where biomass retention may constitute the rate-limiting step.

AD sludge, an important biosolid generated in WWTPs, meets only the Class B stabilization requirements as per U.S. EPA guidelines without thermal treatment (Boczek et al., 2023). Upgrading it to Class A biosolids could substantially expand its reuse potential in areas like in lawns, parks, roadsides, thereby significantly reducing the transportation costs associated with sludge disposal (U.S. Environmental Protection Agency). Prior studies have shown that aerobic post-treatment can also enhance the removal of other contaminants from AD sludge, including heavy metals (Wang et al., 2024, 2021c), pharmaceuticals, personal care products, and per- and polyfluoroalkyl substances (Li et al., 2022). The efficacy of ABAD process in removing these contaminants need to be evaluated.

The VS loading rate of ∼ 6 kg/m^3^/d achieved with our laboratory setup does not represent an upper limit of this process. The deteriorated performance at a VS loading rate of 6.7 kg/m^3^/d in Phase VI was an artefact of the limited capacity of the laboratory aeration system, which is far lower than what achievable in practice. At an HRT of 3 days in Phase VI, the DO in bulk liquid was only 0.5 ± 0.3 mg L^-1^. It was previously reported that *Ca.* Nitrosoglobus has an apparent affinity constant (*K_o_*) of ∼ 1 mg O_2_ L^-1^ in suspended culture (Wang et al., 2021b). The *K_o_* should be even higher for the biofilm culture used in this study. Therefore, a bulk DO concentration of 0.5 ± 0.3 mg L^-1^ mg L^-1^ should have subjected *Ca.* Nitrosoglobus to severe limitation of substrate (O_2_). The aeration system was not optimized in the present study due to the scale limitation. A higher VS loading rate may be achievable at a larger-scale in future through engineering optimization.

The ABAD process slightly improved the sludge dewaterability, as indicated by CST and SRF (Fig. 5). However, no significant change in the solids contents of dewatered cake were detected, which could be due to scale limitation. This issue needs to be further investigated in up-scaling studies. The improved dewaterability may be attributable to the acidic pH. It has been reported that acidic conditions promote sludge aggregation via neutralizing negatively charged functional groups on sludge surface associated with extracellular polymeric substances (EPS) (Cai et al., 2018; Yu et al., 2022). Future studies could validate this by examining the molecular structures of EPS (through spectroscopic approaches) and their correlations with sludge characteristics such as dewaterability, settleability (e.g., dewaterability, settleability) (Guo et al., 2024; Yu, 2020).

The ABAD process has gas emissions, with 1.3% and 2.7% of the nitrogen in the feed sludge emitted as NO and N_2_O, respectively (Fig. S3). These values are higher than conventional aerobic digester operated at neutral pH range (Novak et al., 2011; Parravicini et al., 2008; Tomei et al., 2011; Wang et al., 2021a), and comparable to previous acidic aerobic digester treating secondary sludge (Duan et al., 2018; Lu et al., 2023). NO emission could be due to chemical decomposition of nitrite, which was stimulated at acidic pH (Lu et al., 2023; Udert et al., 2005). The high N_2_O emission was likely attributed to either the inhibition of N_2_O reductase at acidic pH (Blum et al., 2018) or dimerization of HNO (2HNO → N_2_O + H_2_O) (Johnson and Hornstein, 2003). NO is a hazardous gas while N_2_O is a potent greenhouse gas. Similar off gas emissions issues have also been reported for ATAD process, in the forms of NH_3_ and reduced sulfur compounds (Shanchayan, 2006). As such, the ATAD reactors are usually sealed, with the off gases treated in biofilters before venting. Likewise, the off gas of ABAD may also need to be treated using biofilters where denitrifies can convert NO and N_2_O to N_2_ (Jiang et al., 2008).

Apart from NO and N_2_O, still about 50% of influent nitrogen was lost via unidentified pathways (Fig. 2). The most probable pathway is denitrification to N_2_, which occurred in the anoxic zones within the biofilm and thick sludge flocs, although other physicochemical processes cannot be excluded. For instance, NO may undergo further oxidization, producing gases like NO_2_ and N_2_O_3_ (Udert et al., 2005), which were not measured in this study. Future study needs to clarify the detailed mechanisms of nitrogen loss.

It is to be noted that the process demonstrated in this study should not be limited to treat AD sludge, but should also be able to intensify aerobic digestion of other sludge sources, e.g., primary or secondary sludge, although the oxygen limitation issue encountered in this study may be even more severe. It remains an interesting research question to investigate to which extent the capacity of an aerobic digester receiving primary/secondary sludge can be increased with the ABAD process. Besides, the DO and FNA concentrations were not optimized in the present study and could be explored in future.

## Conclusions

○ An auto-acidifying biofilm-based aerobic digestion (ABAD) process was successfully established and operated for 450 days. This is the first application of MBBR configuration for sludge treatment.
○ The ABAD process achieved the highest level of sludge stabilization (Class A) and a sludge reduction efficiency of 27.4 ± 5.2%, within a short retention time of only 3.5 days at ambient temperatures. Meanwhile, dewaterability was slightly improved.
○ The ABAD process offers a promising solution for intensifying sludge treatment systems. The intensification is a cascade effect of the MBBR carriers and the acidity/FNA. The carriers effectively retain acid tolerant AOB, which generates acidic pH and FNA. The acidity/FNA, in turn, directly enhances capacity by accelerating sludge hydrolysis and pathogen inactivation.

## Materials and methods

### Sources of acid tolerant AOB culture, and AD sludge

Two acid-tolerant AOB *Ca.* Nitrosoglobus cultures, one in carrier-biofilm and one in suspended sludge form, were used to seed the MBBR and the SS digester. The carriers originated from a lab-scale MBBR treating low-alkalinity municipal wastewater, with an ammonia removal efficiency of over 80%, respectively (Meng et al., 2022). The MBBR operated at pH 4.2. The biofilm on the carriers contained *Ca.* Nitrosoglobus, as the only detected AOB, with a relative abundance of 16% according to amplicon sequencing analysis (Meng et al., 2022).

The suspended Nitrosoglobus-containing culture originated from a lab-scale parent reactor fed with low-alkalinity municipal wastewater (Wang et al., 2021e). The parent reactor converted ammonia to nitrite, with influent and effluent ammonia concentrations of 91.7 ± 9.3 and 15.4 ± 3.7 mg N L^-1^, respectively. Due to ammonia oxidation and inadequate alkalinity in the feed, the parent reactor operated within an acidic pH range of 4.5–5.0. The parent reactor had a mixed liquor suspended sludge (MLSS) concentration of 1.5 ± 0.1 g L^-1^. The sludge contained *Ca.* Nitrosglobus as the only detectable AOB, with a relative abundance of 1.9 ± 0.1%.

The feed sludge, i.e., AD sludge, was collected from a full-scale anaerobic digester in a local WWTP (Brisbane, Australia). The AD sludge was collected every two weeks, transported to the lab within an hour, and kept refrigerated at 4 °C before being fed to the digesters. The main characteristics of the AD sludge are listed in Table S1.

### Set-up and operation of the MBBR and the SS digesters

Two aerobic digesters (referred to as MBBR digester and SS digester, respectively), each with a working volume of 1.5 L, were set up to treat the AD sludge. The MBBR digester was seeded with 540 mL pre-colonized carriers (the carrier volume was measured with a measuring cylinder without the addition of water) and then was filled with Milli-Q water until the total volume reached 1.5 L, giving a filling ratio of 36%. The SS digester was inoculated with 500 mL Nitrosoglobus-containing mixed sludge liquor and 1000 mL Milli-Q water. Such inoculation strategies gave both aerobic digesters a volumetric ammonium removal rate of ∼ 60 mg N L^-1^ d^-1^, calculated based on the volumetric rates of the their parent reactors. The two digesters were operated in an air-conditioned laboratory with the temperature controlled at 22 ± 1 °C.

The sludge discharge/feeding took place at 12pm every day. A pre-designed amount of sludge was first discharged from the digesters, followed by the feeding of the same volume of AD sludge. The volume of sludge exchanged in each cycle varied with the operational phases, calculated based on the HRT applied in these phases (Table 1). For example, 100 mL sludge was exchanged every day in phase I when an HRT of 15 days was applied. Before each sludge discharging event, the digester was replenished to 1.5 L with Milli-Q water to compensate for any water loss.

The digesters were constantly mixed with magnetic stirrers at 250 rpm, and were aerated with compressed air at an air flowrate of 3.5 L min^-1^ via an air stone. This flowrate is the maximum achievable with the laboratory aeration pump without causing foam overflow from the reactor. The DO was monitored with an optical DO sensor (inPro 6960i, Mettler Toledo) and a multiparameter transmitter (M800, Mettler Toledo), but not controlled. The pH was monitored with a pH electrode (general-purpose pH probe, TPS) and a transmitter (mini CHEM, TPS), but not controlled.

The MBBR digester was operated for 450 days, consisting of six phases. From Phase Ⅰ to Phase Ⅵ, the HRT was shortened stepwise from 15 days to 3 days (Table 1). The operational conditions were otherwise the same among these phases. The SS digester was operated only for 50 days, comprising two phases. In Phase Ⅰ (Day 0–30), the SS digester was operated the same way as the MBBR digester. In Phase Ⅱ when the HRT was decreased to 10 days, the pH of the SS digester rapidly increased to ∼ 8, signalling the termination of microbial ammonia oxidation. The SS digester was terminated thereafter. Various liquid/solids phase parameters were monitored regularly via manual sampling, as described in Table S2.

### Nitrogen (N) and phosphorus (P)

The concentrations of ammonium (NH_4_^+^), nitrite (NO_2_^-^), nitrate (NO_3_^-^) were measured with a flow injection analyser (Lachat QuickChem8000, Milwaukee, WI). For the measurement of total Kjeldahl nitrogen (TKN) and total phosphorus (TP), unfiltered sludge samples were digested first, which converted organic ammino N and phosphate to NH_4_^+^ and to PO_4_^-^, respectively. It should be noted that the Kjeldahl method cannot account for organic N in the forms of azide, azine, azo, oxime, which are expected to be present in very low concentrations in domestic sludge.

### TS and VS

The concentrations of TS and VS were measured following the standard methods (American Public Health Association, 2005). The sludge reduction efficiencies of TS and VS were calculated as, with TS as an example, (TS_in_ – TS_out_) / TS_in_ *100, where TS_in_ and TS_out_ represent the TS in the feed and discharged sludge, respectively.

### Specific oxygen uptake rate (SOUR)

SOUR was calculated as the quotient of the oxygen uptake rate (OUR) and the VS concentration. The OUR was measured following the protocols described in *Supporting information (SI)*.

### Dewaterability

The dewaterability was evaluated using three indices, i.e., the solid contents in the dewatered sludge cake obtained via centrifugation, CST, and SRF, with the detailed procedures presented in SI.

### DNA extraction, amplicon sequencing, and data analysis

DNA was first extracted from the both the suspended sludge and biofilm samples. The biomass on carriers was scratched into a 1.5 mL sterilized Eppendorf tube before DNA extraction. The extracted DNA was subsequently subject to 16S rRNA genes amplicon sequencing. The sequenced libraries were processed in quantitative insights into microbial ecology II (QIIME II). Details of these can be found in SI.

### Faecal Coliforms measurement

*Faecal Coliforms* was chosen as the indicator microorganism of pathogenic microbes. The *Faecal Coliforms* abundances in the feed/discharged sludges were assessed using Colilert®−18 Test kit (IDEXX laboratories, Australia) following the instructions from the manufacture. First, sludge samples were diluted to 100 mL using sterilized Milli-Q water. Dilution factors ranging from 10^2^ to 10^5^ times were chosen to ensure the pathogen concentration was within the detection limit of the test kit (i.e., 1–2420 cells per 100 mL). Subsequently, the diluted sludge samples were mixed with Colilert®−18 reagents. The mixture was transferred into a Quanti-Tray® 2000. This tray was then sealed in the Quanti-Tray sealer (IDEXX laboratories, Australia), before being incubated at 45 ± 1 °C and 120 rpm for 18 h. The number of positive wells (yellow) on the Quanti-Tray was counted and recorded, through which the most probable number (MPN) of *Faecal Coliforms* was estimated via consulting the Quanti-Tray®/2000 MPN Table (IDEXX laboratories, Australia).

### Statistical analyses

Statistical significance was evaluated by two-tailed *t*-test. In this study, the *p*-values less than 0.001, 0.01, 0.05 were summarized with three, two, and one asterisks, respectively. Variability in input variables is expressed as standard deviation, and this is explicitly stated where relevant. Confidence in output is expressed as standard error (±).

## CRediT authorship contribution statement

**Zhiyao Wang:** Writing – review & editing, Writing – original draft, Methodology, Investigation, Formal analysis, Data curation, Conceptualization. **Xi Lu:** Investigation, Data curation. **Min Zheng:** Writing – review & editing, Conceptualization. **Zhetai Hu:** Resources, Investigation. **Damien Batstone:** Writing – review & editing, Funding acquisition. **Zhiguo Yuan:** Writing – review & editing, Funding acquisition. **Shihu Hu:** Writing – review & editing, Supervision, Project administration, Funding acquisition.

## Declaration of competing interest

The authors declare that they have no known competing financial interests or personal relationships that could have appeared to influence the work reported in this paper.

## Data Availability

Data will be made available on request

## Appendix Supplementary materials

Image, application 1
