# Supplementary material for: Quadrupling the capacity of post aerobic digestion treating anaerobically digested sludge using a moving-bed biofilm (MBBR) configuration
Source: Water Res X. 2024 Jul 23;24:100240. doi: 10.1016/j.wroa.2024.100240 (PMC11347825; doi:10.1016/j.wroa.2024.100240)
Supplement: Supplementary file 1 [file mmc1.docx]

***Supplementary Materials***

**Quadrupling the capacity of post aerobic digestion treating anaerobically digested sludge using a moving-bed biofilm (MBBR) configuration**

Zhiyao Wang 1^, ⊥^, Xi Lu 1, ^⊥^, Min Zheng 2, *, Zhetai Hu 1, Damien Batstone 1, Zhiguo Yuan 3, Shihu Hu 1, *

^1^ Australian Centre for Water and Environmental Biotechnology (ACWEB, formerly AWMC), The University of Queensland, St. Lucia, Queensland 4072, Australia

^2^ Water Research Centre, School of Civil and Environmental Engineering, University of New South Wales, Sydney, New South Wales 2052, Australia

^3^ School of Energy and Environment, City University of Hong Kong, Hong Kong SAR, China

**Corresponding author**

Email address: [s.hu@uq.edu.au](mailto:s.hu@uq.edu.au); [min.zheng1@unsw.edu.au](mailto:min.zheng1@unsw.edu.au)

**The following are included as supporting information for this paper:**

Number of pages: 10

Number of figures: 5

**S1 Characteristics of the feed sludge**

**Table S1.** Main characteristics of anaerobically digested sludge used in this study.

| Parameters | Mean ± Standard deviation |
| --- | --- |
| Total solids (TS) (g L^-1^) | 28.9 ± 0.9*_n_* _= 60_ |
| Volatile solids (VS) (g L^-1^) | 20.1 ± 3.7 *_n_* _= 60_ |
| Total chemical oxygen demand (TCOD) (g L^-1^) | 32.5 ± 4.9*_n_* _= 10_ |
| Total Kjeldahl nitrogen (TKN) (g N L^-1^) | 3.4 ± 0.1*_n_* _= 10_ |
| Total phosphorus (TP) (g P L^-1^) | 0.6 ± 0.1*_n_* _= 10_ |
| Ammonium (g NH_4_^+^-N L^-1^) | 1.2 ± 0.1*_n_* _= 30_ |
| Phosphate (mg PO_4_^3-^-P L^-1^) | 132.0 ± 38.0*_n_* _= 30_ |
| pH | 7.8 ± 0.2*_n_* _= 63_ |

**S2 Analytical methods**

***Chemical oxygen demand (COD)***

The concentrations of COD were measured following the standard methods American Public Health Association, 2005). Total COD (TCOD) was determined using the unfiltered sludge samples, while the soluble COD (SCOD) was determined on the supernatant obtained via filtration using the disposable MF-Millipore™ membrane filters with pore sizes of 0.22 𝜇m.

**Table S2.** Summary of the monitoring protocols

| Parameters | | Frequency |
| --- | --- | --- |
| Nitrogen and phosphorus | Total Kjeldahl nitrogen (TKN) | Once per week |
|  | Total phosphorus (TP) |  |
|  | Ammonium (NH_4_^+^) | 2–3 times per week |
|  | Nitrite (NO_2_^-^) |  |
|  | Nitrate (NO_3_^-^) |  |
|  | Phosphate (PO_4_^-^) |  |
|  | Total inorganic nitrogen (TIN, NH_4_^+^+ NO_2_^-^+ NO_3_^-^) |  |
|  | Total nitrogen (TN, TKN+ NO_2_^-^+ NO_3_^-^) |  |
| Chemical oxygen demand (COD) | Total COD | Once every two weeks |
|  | Soluble COD |  |
| Solids reduction | Total solids (TS) | 2–3 times per week |
|  | Volatile solids (VS) |  |
| Sludge stabilization | *Faecal Coliforms* | Once per week (steady state only) |
|  | Specific oxygen uptake rate (SOUR) |  |
| Dewaterability | Solids contents in the dewatered sludge cake obtained with centrifugation | Twice per week (in Phase V steady state) |
|  | Capillary suction time (CST) |  |
|  | Specific resistance to filtration (SRF) |  |
| Microbial community | Samples stored for amplicon sequencing | Once per month |

***Specific oxygen uptake rate (SOUR)***

SOUR was calculated as the quotient of the oxygen uptake rate (OUR) and the VS concentration. The OUR was measured in an *ex situ* respirometer, which comprised a 1 L reactor and an optical DO electrode (METTLER TOLEDO, Switzerland). The DO electrode was connected to a computer for data acquisition via a programmable logic controller (PLC).

Each sludge sample was consecutively measured for three times. For each measurement, 100 mL sludge was collected and diluted to 1L with 900 mL of Milli-Q water. The diluted sludge was aerated with compressed air in a 2 L beaker till DO was above 7 mg L^-1^. Afterwards the aerated sludge was injected to the respirometer, leaving no headspace. The DO concentration profile was automatically recorded at an interval of 0.5 second until DO < 5 mg L^-1^. The OUR was determined by the linear regression of the DO profile.

***Dewaterability***

Polymer (Polymeric aluminum chloride (PAC) as polymer (7.89 g/L)) was added to a 25 mL sludge sample. The polymer was dosed, while mixed and stirred, until coagulation occurred. The volume of polymer added was recorded. Once coagulated, the sludge was drained into solids cake through a belt filter fabric. The solids cake was subsequently centrifuged in a centrifuge cup with the belt filter fabric at 3750 rpm for 10 min. The solids content (%) of the cake was determined after discharging the supernatant.

Capillary suction time (CST) was measured with a CST instrument (304M, Triton, UK) equipped with a 10 mm diameter funnel. Specific resistance to filtration (SRF) was measured using the method described in Liu et al. (2012).

The dewaterability was evaluated using three indices, i.e., the solid contents in the dewatered sludge cake, the capillary suction time (CST), and the specific resistance to filtration (SRF). The dewaterability was measured following the procedures reported by Yu et al.

***DNA extraction & Amplicon sequencing & Data analysis***

DNA was extracted from the biomass samples using FastDNA^®^ spin kit (MP Biomedicals, Santa Ana, CA) following the protocols provided by manufacturer. The quality of the extracted DNA was checked using gel electrophoresis and the concentration of the extracted DNA was measured by a NanoDrop spectrophotometer (NanoDrop Technologies, Wilmington, DE, USA).

Amplicon sequencing was conducted on the extracted DNA at the Australian Centre for Ecogenomics (Brisbane, Australia). Firstly, the 16S rRNA genes library was prepared as described in the workflow outlined by Illumina. Then the V3–V4 regions of the 16S rRNA genes was amplified using modified universal primers 338F (5’- ACT CCT ACG GGA GGC AGC AG -3’) and 806R (5’- GGA CTA CHV GGG TWT CTA AT - 3’). The amplifications were performed via polymerase chain reaction (PCR) in NEBNext^®^ UltraTM II Q5^®^ Mastermix (New England Biolabs) under standard PCR conditions. The PCR amplicons were afterwards purified using Agencourt AMPure XP beads (Beckman Coulter). Subsequently, the purified amplicons were indexed with unique 8bp barcodes using the Illumina Nextera XT 384 sample Index Kit A-D (Illumina FC-131-1002). Lastly, the indexed amplicons were pooled together in equimolar concentrations and sequenced on a MiSeq Sequencing System (Illumina) in paired end mode as specified in the user’s protocols.

The sequenced libraries were processed in quantitative insights into microbial ecology II (QIIME II). Primer sequences and poor-quality sequences were trimmed to 250 bases, and any with less than 250 bases was excluded. After quality control, operational taxonomic units (OTUs) were clustered at 97% identity using the Markov Cluster algorithm. Clustered representative sequences were taxonomically assigned against the Silva database ^11^. The percentage of captured diversity was assessed by the Good's coverage estimator ^12^.

**S3 Long-term N conversion performance in the SS digester**

**Figure S1** The temporal profiles of (A) pH; (B) nitrite concentration; and (C) free nitrous acid (FNA) concentration in the SS digester. The hydraulic retention time (HRT) was decreased from 15 days in Stage Ⅰ to 10 days in Stage Ⅱ.

**S4 Performance of sludge reduction and stabilization in the SS digester**

***The SS digester***

**Figure S2** Comparisons of (A) total solids concentration; (B) volatile solids concentration; (C) *Faecal Coliform* (indicators of pathogenic microorganism) concentration; and (D) specific oxygen uptake rate (SOUR) between the feed sludge (i.e., anaerobically digested sludge) and the discharged sludge across Stage I–II. Error bars represent standard deviations.

**S4 NO and N_2_O emissions from the MBBR digester**

**Figure S3** The profiles of pH, off-gas NO and N_2_O concentrations in a typical cycle of the MBBR digesters in Phase V. Sludge feeding occurred at 0 h.

**S5 Microbial community compositions of the biofilm and the suspended sludge from the MBBR digester**

**Figure S4** Microbial compositions of the suspended sludge and the biofilm at the Phylum level. Samples was taken from the MBBR digester in Phase V.

**Figure S5** Microbial compositions of the suspended sludge and the biofilm at the Genus level. Samples was taken from the MBBR digester in Phase V.

**S6 Comparison between this study and autothermal aerobic digestion (ATAD)**

**technology**

**Table S3.** Technology comparison between the auto-acidifying aerobic digestion technology and the autothermal aerobic digestion technology.

|  | **Auto-acidifying aerobic digestion** | | **Autothermal aerobic digestion** |
| --- | --- | --- | --- |
| **Configurations** | Suspended sludge-based^1–4^ | Biofilm-based (this study) | Suspended sludge-based^5–9^ |
| **Temperature (°C)** | 22 ± 1 °C | | 40-80 °C |
| **pH** | 4–5 | | 7–8 |
| **Sludge sources** | Secondary sludge, anaerobically digested sludge | Anaerobically digested sludge | Primary sludge, secondary sludge, animal/human wastes, etc. |
| **Aeration** | Air | | Air or pure oxygen |
| **HRT (days)** | 13–15 | 3.5 | 3–15 |
| **Volatiles solids loading rate (kg/m^3^/d)** | 1.0–2.0 | 5.7 | 1.0–8.0 |
| **Volatile solids reduction efficiency** | 20%–30% | 25–30% | 30–60% |
| **Pathogen inactivation** | Grade A or B biosolids | Grade A biosolids | Grade A biosolids |
| **Toxic metal removal** | With the potential to solubilize toxic metals. Require pH < 3. Demonstrated in batchwise study only.^4^ | | Not reported |
| **Capital requirement** | Anti-corrosion measures are potentially needed. Sealed reactor is potentially needed, to control foaming and off gas emissions. | | Proper insulation and sealed reactor are required to maintain temperatures, to control foaming, and to avoid odour issue. |
| **Dewaterability** | Not reported | Slightly improved | Deteriorated |
| **Off-gas issue** | NO, N_2_O, etc. Off gas treatment is needed. | | Odour (NH_3_ and reduced sulfur compounds). Off gas treatment is needed. |
| **Demonstration scale** | Laboratory; Pilot on going | Laboratory | Laboratory; Pilot; and Full-scale |

**References**

(1) Lu, X.; Wang, Z.; Duan, H.; Wu, Z.; Hu, S.; Ye, L.; Yuan, Z.; Zheng, M. Significant Production of Nitric Oxide by Aerobic Nitrite Reduction at Acidic PH. *Water Res* **2023**, *230*, 119542.

(2) Duan, H.; Ye, L.; Lu, X.; Batstone, D. J.; Yuan, Z. Self-Sustained Nitrite Accumulation at Low PH Greatly Enhances Volatile Solids Destruction and Nitrogen Removal in Aerobic Sludge Digestion. *Environ Sci Technol* **2018**, *53* (3), 1225–1234.

(3) Wang, Z.; Zheng, M.; Duan, H.; Ni, G.; Yu, W.; Liu, Y.; Yuan, Z.; Hu, S. Acidic Aerobic Digestion of Anaerobically-Digested Sludge Enabled by a Novel Ammonia-Oxidizing Bacterium. *Water Res* **2021**, *194*, 116962.

(4) Wang, Z.; Ni, G.; Xia, J.; Song, Y.; Hu, S.; Yuan, Z.; Zheng, M. Bioleaching of Toxic Metals from Anaerobically Digested Sludge without External Chemical Addition. *Water Res* **2021**, *200*, 117211.

(5) Layden, N. M.; Mavinic, D. S.; Kelly, H. G.; Moles, R.; Bartlett, J. Autothermal Thermophilic Aerobic Digestion (ATAD) — Part I: Review of Origins, Design, and Process Operation. *Journal of Environmental Engineering and Science* **2007**, *6* (6), 665–678. https://doi.org/10.1139/S07-015.

(6) Layden, N. M.; Kelly, H. G.; Mavinic, D. S.; Moles, R.; Bartlett, J. Autothermal Thermophilic Aerobic Digestion (ATAD) — Part II: Review of Research and Full-Scale Operating Experiences. *Journal of Environmental Engineering and Science* **2007**, *6* (6), 679–690. https://doi.org/10.1139/S07-040.

(7) Matsch, L. C.; Drnevich, R. F. Autothermal Aerobic Digestion. *J Water Pollut Control Fed* **1977**, 296–310.

(8) Kelly, H. G.; Melcer, H.; Mavinic, D. S. Autothermal Thermophilic Aerobic Digestion of Municipal Sludges: A One‐year, Full‐scale Demonstration Project. *Water Environment Research* **1993**, *65* (7), 849–861.

(9) Gould, M. S.; Drnevich, R. F. Autothermal Thermophilic Aerobic Digestion. *Journal of the environmental engineering division* **1978**, *104* (2), 259–270.

(10) Anthonisen, A. C.; Loehr, R. C.; Prakasam, T. B. S.; Srinath, E. G. Inhibition of Nitrification by Ammonia and Nitrous Acid. *Journal of the Water Pollution Control Federation* **1976**, *48* (5), 835–852. https://doi.org/10.1016/0168-6496(92)90072-2.

(11) Quast, C.; Pruesse, E.; Yilmaz, P.; Gerken, J.; Schweer, T.; Yarza, P.; Peplies, J.; Glöckner, F. O. The SILVA Ribosomal RNA Gene Database Project: Improved Data Processing and Web-Based Tools. *Nucleic Acids Research* **2013**, *41* (D1), D590–D596. https://doi.org/10.1093/nar/gks1219.

(12) Good, I. J. The Population Frequencies of Species and the Estimation of Population Parameters. *Biometrika* **1953**, *40* (3–4), 237–264. https://doi.org/10.2307/2333344.
